# Convenient Preparation, Thermal Properties and X-ray Structure Determination of 2,3-Dihydro-5,6,7,8-tetranitro-1,4-benzodioxine (TNBD): A Promising High-Energy-Density Material

**DOI:** 10.3390/ijms25105099

**Published:** 2024-05-07

**Authors:** Jonas Šarlauskas

**Affiliations:** Department of Xenobiotics Biochemistry, Institute of Biochemistry of Vilnius University, Sauletekio 7, LT-10257 Vilnius, Lithuania; jonas.sarlauskas@bchi.vu.lt

**Keywords:** 1,4-benzodioxane, nitration, nitro derivatives, tetranitro, synthesis, X-ray crystallography, structure, thermal analysis, high-energy high-density material (HEDM)

## Abstract

2,3-dihydro-5,6,7,8-tetranitro-1,4-benzodioxine (TNBD), molecular formula = C_8_H_4_N_4_O_10_, is a completely nitrated aromatic ring 1,4-benzodioxane derivative. The convenient method of TNBD synthesis was developed (yield = 81%). The detailed structure of this compound was investigated by X-ray crystallography. The results of the thermal analysis (TG) obtained with twice re-crystallized material revealed the onset at 240 °C (partial sublimation started) and melting at 286 °C. The investigated material degraded completely at 290–329 °C. The experimental density of 1.85 g/cm^3^ of TNBD was determined by X-ray crystallography. The spectral properties of TNBD (NMR, FT-IR and Raman) were explored. The detonation properties of TNBD calculated by the EXPLO 5 code were slightly superior in comparison to standard high-energy material—tetryl (detonation velocity of TNBD—7727 m/s; detonation pressure—278 kbar; and tetryl—7570 m/s and 226.4 kbar at 1.614 g/cm^3^, or 260 kbar at higher density at 1.71 g/cm^3^. The obtained preliminary results might suggest TNBD can be a potential thermostable high-energy and -density material (HEDM).

## 1. Introduction

Aromatic polynitroderivatives are among the most popular high-energy materials (HEMs) [1,2,3,4,5]. From the practical standpoint, HEMs must hold not only high energetic properties but also satisfy other strict requirements [4,5,6,7,8]. The most significant are chemical stability, shock-insensitivity, high density and thermostability [5,9,10,11,12].

The most well-known “old” energetic materials are mostly benzene-derived polynitrocompounds. Later, *N*-heterocyclic high-energy materials (HEMs) began to be considered as perspective candidates for replacing conventional high explosives, such as TNT, RDX, HMX and PETN [9,10,13,14,15].

Although mixed *O*,*N*-heterocycles, such as 1,2,5-, 1,2,4- and 1,3,4-oxadiazoles [16,17,18,19,20], as well as oxadiazole *N*-oxides (furoxans) [21,22,23,24,25,26], are now amongst the most popular newly designed energetic compounds [27], derivatives of “pure” *O*-heterocycles as energetic materials have hardly been mentioned in the scientific literature (except for unstable cyclic peroxides) [28]. 1,4-Benzodioxane (also known as 1,2-ethylenedioxybenzene, benzo-1,4-dioxane (old chemical names) or 2,3-dihydro-1,4-benzodioxin (new systematic name)) is one of many benzene-annulated oxygen *O*-heterocycles [29].

**Figure 1:**
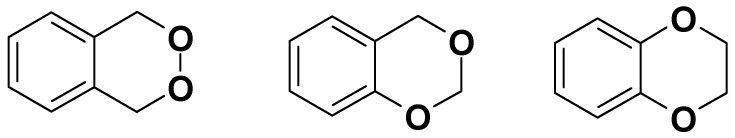
Three structural isomers of benzodioxane (1,2-, 1,3- and 1,4 BD).

It should be noted that there are three known structural isomers of benzodioxane (BD): unstable 1,2-benzodioxane [30,31,32], 1,3-benzodioxane [33] and most stable 1,4-benzodioxane [34] (Figure 1).

It is worth noting that the latest isomer, 1,4-benzodioxane (2,3-dihydro-1,4-benzodioxine), originally synthesized by German chemist Vorländer [35], possessed the highest chemical and thermal stability [36].

Current practical applications of 1,4-benzodioxane include its use in medicinal chemistry and pharmacy, where it serves as an excellent core for structurally diverse pharmacologically active agents [37,38,39].

From 1960 onwards, 1,4-benzodioxane derivatives were extensively synthesized and investigated as biologically active compounds for their wide-spectrum pharmacological activity (e.g., anti-inflammatory, anti-histaminic and local anesthetic, among others) at our University under the direction of Prof. Hab. Dr. Daukšas V. K. (1935–2013) [40,41,42].

Later, in the same laboratory, the nitro and amino derivatives of 1,4-benzodioxane were used as a core structural fragment for the synthesis of novel annulated *O*,*N*-heterocyclic derivatives [43,44,45,46,47].

Surprisingly, nitro derivatives of 1,4-benzodioxane have not received much attention for their prospective use as energetic materials. One of the primary reasons is probably the relatively high cost of 1,4-benzodioxane as a starting material (31.40 EUR per 10 g, according to Merck Company, Rahway, NJ, USA).

In this work, we expand our previous investigation [48] of 1,4-benzodioxane polynitroderivatives and chose one of them, tetranitro-1,4-benzodioxane (TNBD), for a more detailed study of the crystal structure, thermal stability and other properties, with the prospective goal of using it as a thermally stable high-energy high-density material.

The current research focuses on the TNBD compound, an annulated O-heterocycle 1,4-benzodioxan derivative that has been completely nitrated in the aromatic benzene ring.

A more convenient, optimized synthesis of the TNBD substance and its structural characterization via an X-ray crystal diffraction analysis are reported below (Section 3).

## 2. Results and Discussion

### 2.1. Structure and Properties Investigation

#### 2.1.1. Physicochemical Properties

The following are the physicochemical properties: molecular weight—316.14, molecular formula = C_8_H_4_N_4_O_10_, molecular composition (C 30.39% H 1.28% N 17.72% O 50.61%) and oxygen balance OB_CO2_ = −40.48%.

TNBD is a white powder in a dispersed state, colorless prisms in a crystalline state and the crystals are almost insoluble in water and conc. sulfuric acid. TNBD is well soluble and crystallizes from methylacetate, acetone, acetonitrile and 2-butanone but is significantly less soluble in benzene and toluene and almost insoluble in other solvents, such as hexane, dichloroethane or CCl_4_. Many solvents were tested for the TNBD solubility, but only 2-butanone (methylethyl ketone) was found to be the best for growing high-quality crystals suitable for an X-ray diffraction analysis. It is worth noting that TNBD is substantially more hydrolytically stable than analogous structured compounds, such as 1,2,3,4-TetrNB (tetranitrobenzene) and HNB (hexanitrobenzene) (further details are discussed below).

#### 2.1.2. Chemical/Hydrolytic Stability—Preliminary Findings

**Figure 2:**
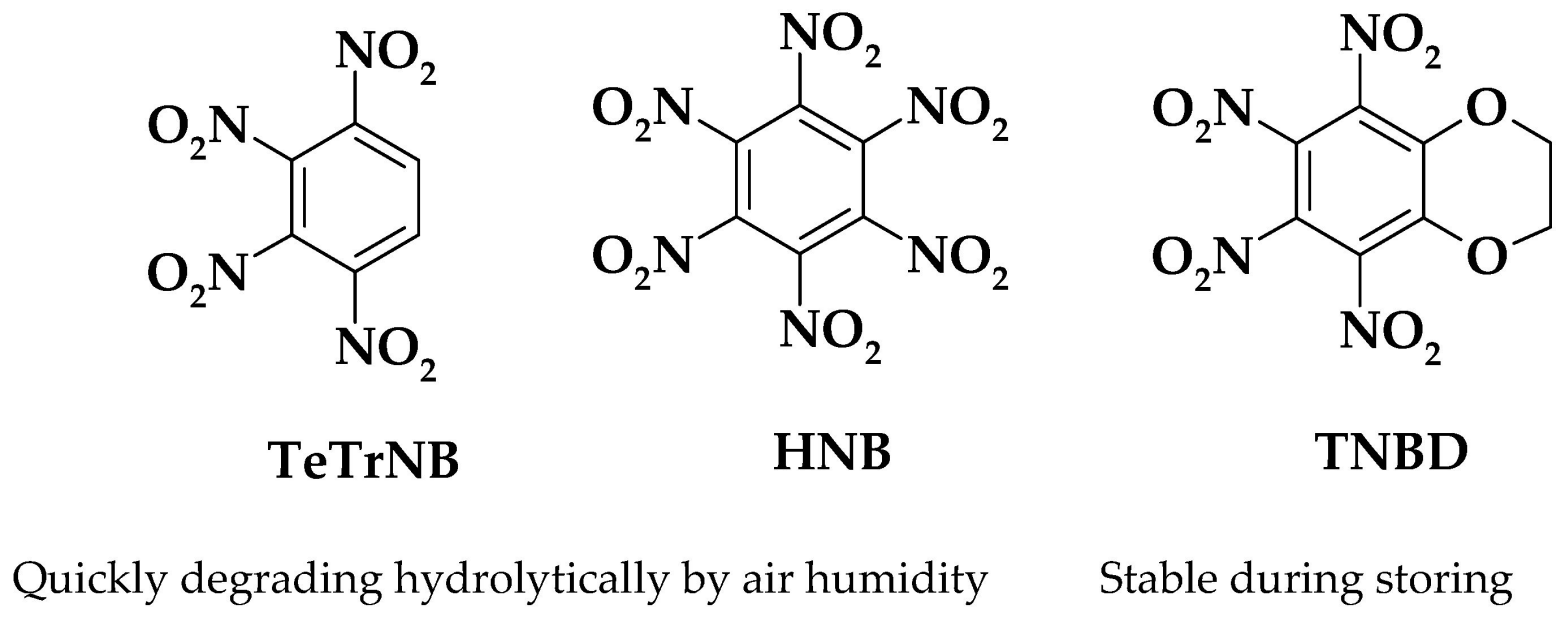
Benzene tetra- and hexanitrocompounds and TNBD stability comparison.

It is generally known that more nitro groups affording benzenes with higher energy suffer from lower chemical stability [49]. Similarly, higher levels of nitration also induce reduced hydrolytic stability. 1,2,3,4-Tetranitrobenzene is an impact-sensitive explosive that easily decomposes in the presence of moisture to 2,4-dinitro-1,3-dihydroxy benzene. Hexanitrobenzene (HNB), another well-known and extremely potent nitroarene explosive [3,5,50], easily hydrolyzes to trinitrophloroglucine when exposed to air humidity [51,52]. Contrary to these HNB properties, the TNBD compound synthesized in this work exhibited good chemical stability under normal conditions (Figure 2).

TNBD is a very chemically stable molecule, which can withstand for a short time conc. sulfuric acid at 100 °C, boiling in the fuming HNO_3_ at 110 °C and exposure to a layer of water (at 20 °C) for one week or much longer. Under normal conditions, TNBD has good hydrolytic stability. Surprisingly, we found no degradation products after 20 years of storing TNBD samples in our laboratory at normal temperature. An additional dioxan ring in the structure of the TNBD molecule appears to have a strong stabilizing effect. Hydrolytic lability, light sensitivity and other undesirable properties of hexanitrobenzene (HNB) limited its practical application [53]. Contrary to these HNB properties, when synthesized in this work, the TNBD compound exhibited good chemical stability under normal conditions. These preliminary findings are based on chromatographic (TLC) and spectral data. No products of degradation or hydrolytic destruction by air humidity were found. An additional experiment, storing the TNBD sample (100 mg) under a water layer (5 cm) for one week (168 h at 20 °C), did not find any analytical changes in the recovered and dried material. However, further and more comprehensive research into TNBD stability is required.

#### 2.1.3. Spectral Properties

FT-IR and Raman confirmed the functional groups of TNBD.

**Figure 3:**
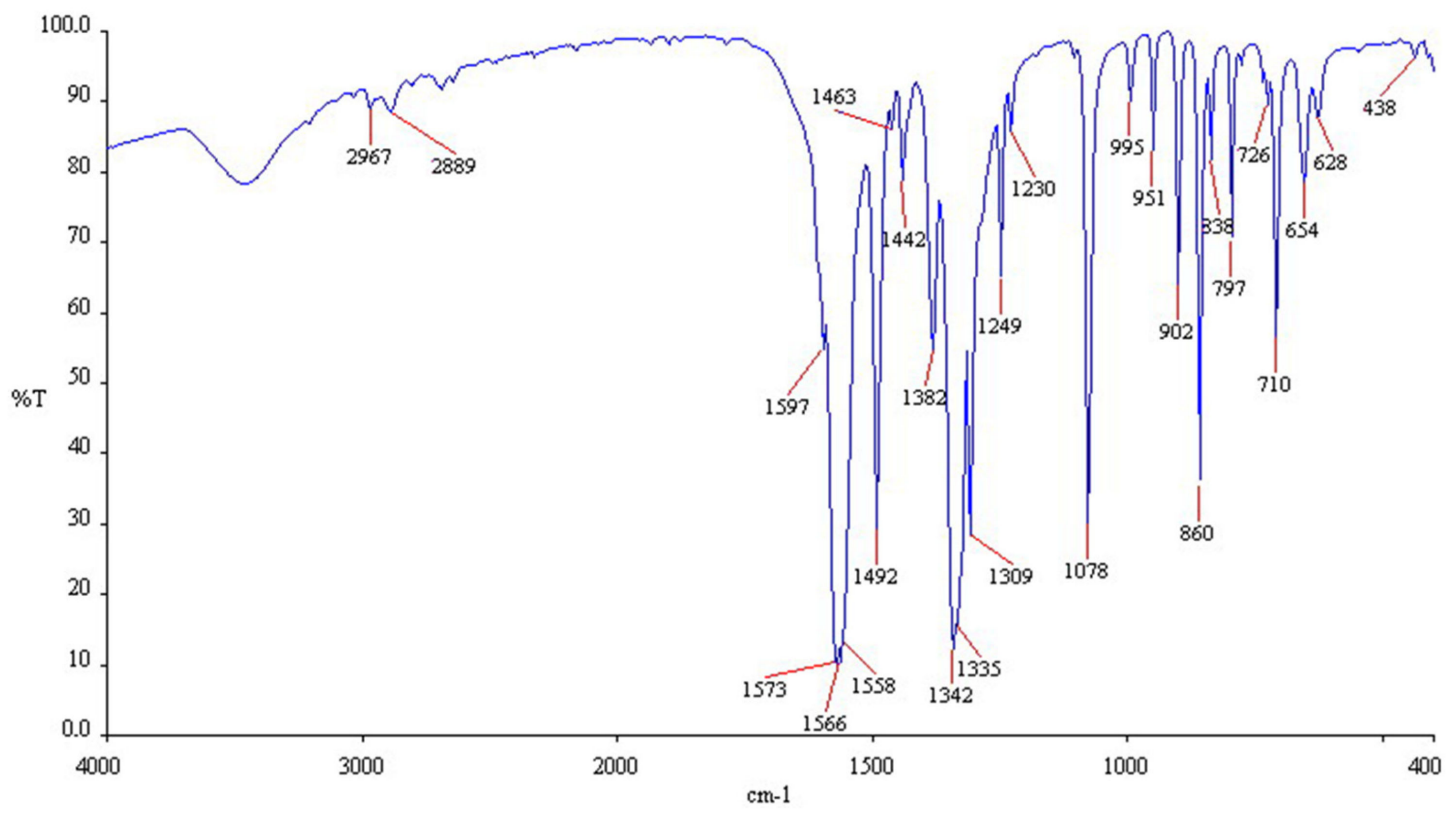
FT-IR spectrum of TNBD registered in KBr disc.

The FT-IR spectra (sample in KBr) displayed two small peaks at 2967 and 2889 cm^−1^, characteristic for a dioxan -CH_2_-CH_2_- fragment (asym. C-H strech) (Figure 3). Two other intense wide bands were typical for aromatic nitro groups: 1566 (sym.) and 1342 cm^−1^ (asym.). An intense peak at 1078 cm^−1^ was a characteristic pattern for two ethereal O-C groups in a dioxane fragment.

The Raman spectrum was characterized by the appearance of very strong bands at 1335 and 1371 cm^−1^ and two weak bands at 3093 and 2981 cm^−1^, which is typical for asym. The C-H stretch in the alkane fragment CH_2_-CH_2_ (dioxane ring) and the medium-intensity band at 1082 cm^−1^ are characteristic for C-O-C groups in a dioxane ring.

**Figure 4:**
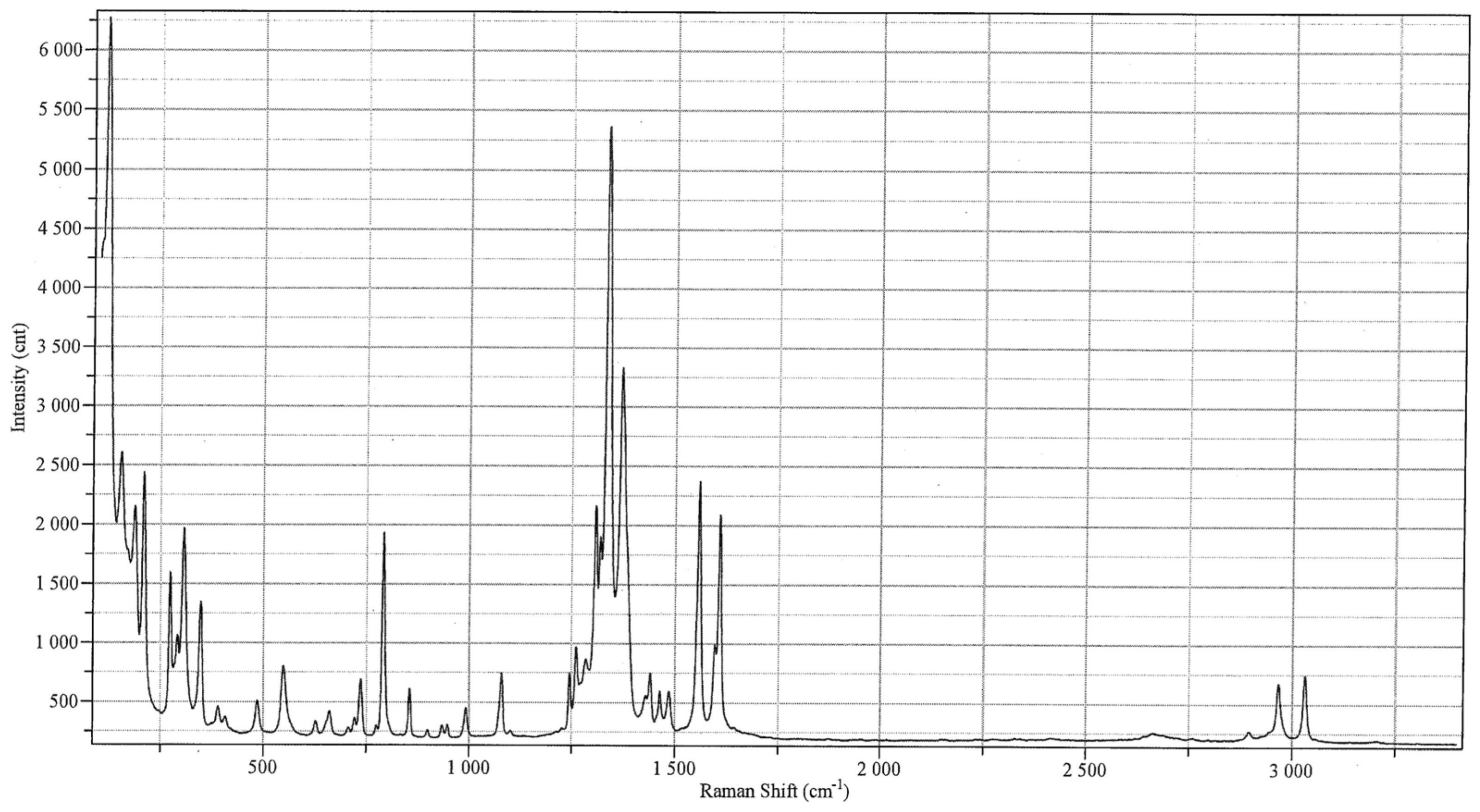
FT-Raman spectrum (100 scans) of TNBD (5,6,7,8-tetranitro-2,3-dihydro-1,4-benzodioxine) powder (cm^−1^): 3020, 2971,1608, 1598, 1559, 1490, 1465, 1441, 1371, 1335, 1318, 1309, 1282, 1258, 1247, 1082, 884, 856, 794, 761, 665, 550, 488, 436, 353, 312, 298 and 280.

The Raman shift spectra measurements (Figure 4) were conducted via excitation with 633 nm. The laser beam was focused to a spot of ca. 1 mm^2^ area (laser power—300 mW).

In the ^1^H spectrum (300 MHz), one singlet at 4.73 was observed (s, 4H, alkane (CH_2_)_2_ fragment) (TNBD molecule is symmetric).

In the NMR ^13^C spectrum (75 MHz), four peaks are described as 143.91 (6- and 7-C); 132.43 (5- and 8-C); 129.02 (2- and 3-C); and 66.31 (CH_2_-CH_2_).

#### 2.1.4. Thermal Properties

**Figure 5:**
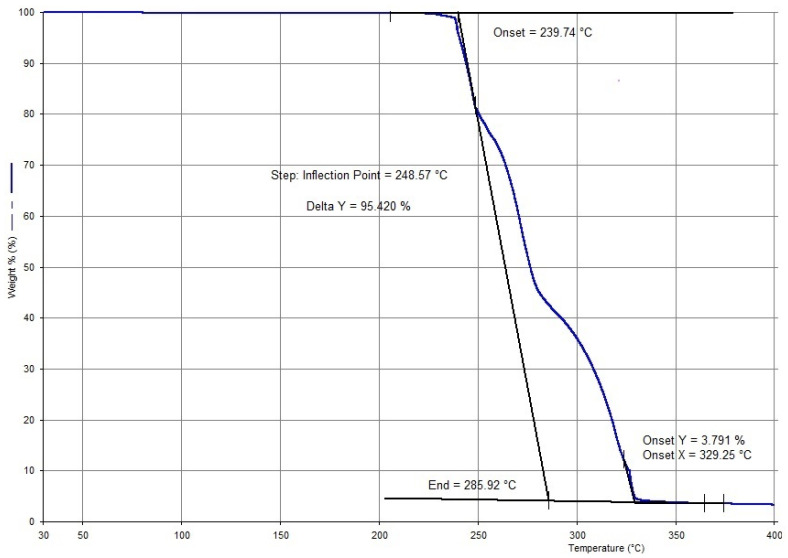
Thermogravimetric analysis (TG, blue line, and onset points are marked by black line) of TNBD sample (10 mg).

An analysis of the thermal properties of TNBD was performed at 10 °C/min and 20 mL/min N_2_ flow rate (Figure 5).

The thermal analysis (TG) of the twice re-crystallized material revealed the onset at 240 °C (partial sublimation started) and melting at 286 °C. The investigated material degraded completely at 290–329 °C, demonstrating that the thermostability of TNBD is close to that of HNS (hexanitrostilbene), also known as thermostable HEM [54].

The thermal stability of TNBD was found to be significantly higher than that of its analogues of a similar structure, 1,2,3,4-tetranitrobenzene (m.p. 120–121 °C) and hexanitrobenzene (HNB) (m.p. 245–262 °C, with decomposition) [51].

#### 2.1.5. Crystal Properties and Structure Factors Leading to High Density

**Figure 6:**
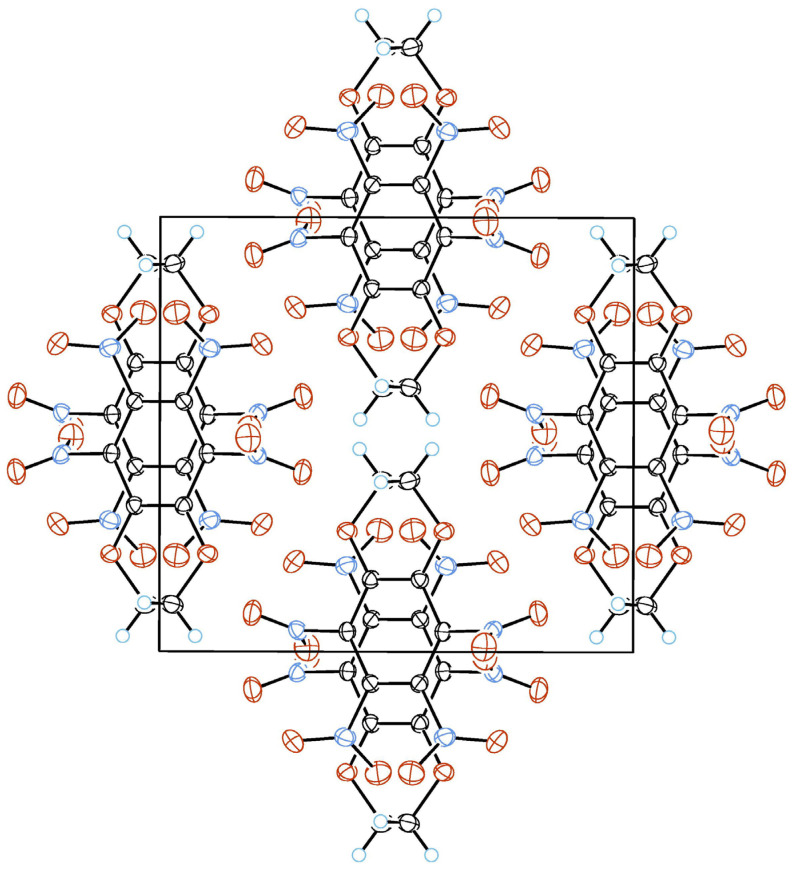
TNBD crystal cell package (OrtepPack). Carbon atoms are represented in black color, nitrogen—in blue and oxygen—in red.

This study was further undertaken to establish the three-dimensional structure and density of TNBD. The crystals of TNBD for its X-ray diffraction were obtained by slow evaporation at room temperature of the solution in 2-butanone. The geometries of TNBD are tabulated below. All the diagrams and calculations were performed using maXus (Bruker Nonius, Delft, The Netherlands and MacScience, Yokohama, Japan). The TNBD crystal cell packing is shown in Figure 6.

**Figure 7:**
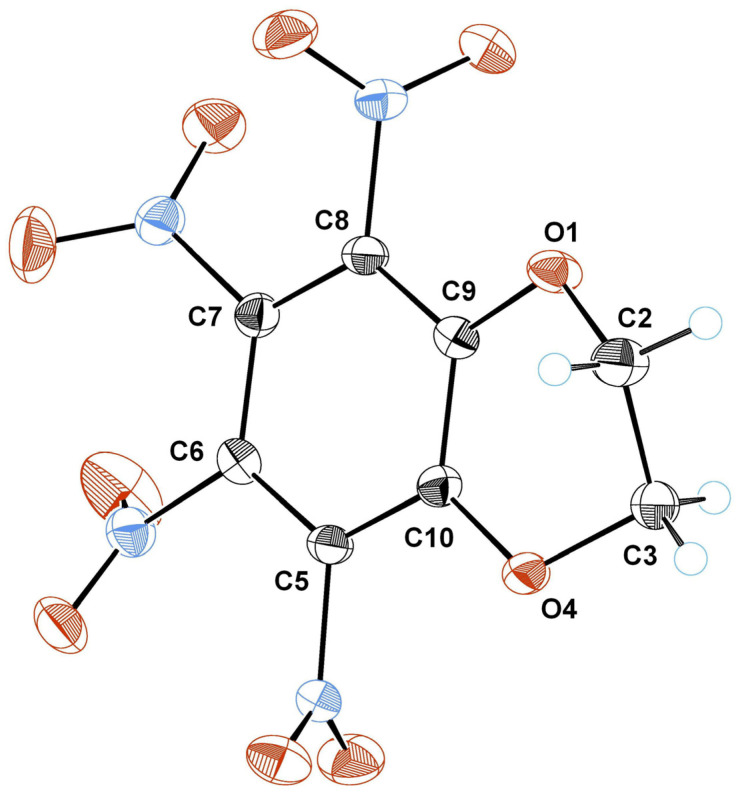
ORTEP representation of TNBD. Carbon atoms are represented in black color, nitrogen—in blue and oxygen—in red. The thermal ellipsoids are presented with 50% probability (CCDC 2320894) (crystal was grown from 2-butanone).

Remarkably, only half of this compound molecule is located in a separate crystal cell unit. The ORTEP representation of the TNBD structure is shown below in Figure 7. The main crystallographic data for TNBD are represented in Table 1.

TNBD—5,6,7,8-tetranitro-2,3-dihydro-1,4-benzodioxine

**Table 1:** The main crystallographic data for TNBD.

Empirical formula	C_8_H_4_N_4_O_10_
Formula weight	316.138
Temperature	173(2) K (−100 °C)
Wavelength	0.71073 Å
Crystal system	Monoclinic
Space group	C 2/c
Unit cell dimensions	a = 10.9946(4), A alpha = 90.0 deg. b = 10.1532(4), A beta = 94.415(2) deg. c = 10.1814(3), A gamma = 90.0 deg.
Volume	1133.18(7) Å^3^
Z	4
Density (calculated)	1.853 g/cm^3^
Density (measured, pycnom.)	1.84 g/cm^3^
Absorption coefficient	0.176 mm^−1^
F(000)	640
Crystal size	0.31 × 0.22 × 0.21 mm
Two theta max. for data	58.0 deg.
Reflections collected	2368
Independent reflections	1464 [R(int) = 0.020]
Refinement method	Full-matrix least-squares on F^2^
Data/restraints/parameters	1292/0/108
Final R indices [I > 3sigma(I)]	R_1_ = 0.032, wR_2_ = 0.077
R indices (all data)	R_1_ = 0.041, wR_2_ = 0.116
Largest diff. peak and hole	0.41 and −0.37 e.Å^−3^

Full geometry table (Table S1) for the compound TNBD are presented below in Supplementary Materials.

It is worth noting that the nitro groups in positions 7 and 8 of the TNBD are twisted out of the benzene ring plane by different angles, 46.143 and 68.706 degrees (because of symmetry, similar angles are observed for another two NO_2_ groups in positions 5 and 6, correspondingly).

For comparison, in HNB (hexanitrobenzene), the nitro groups are rotated at 53° [50,51].

**Figure 8:**
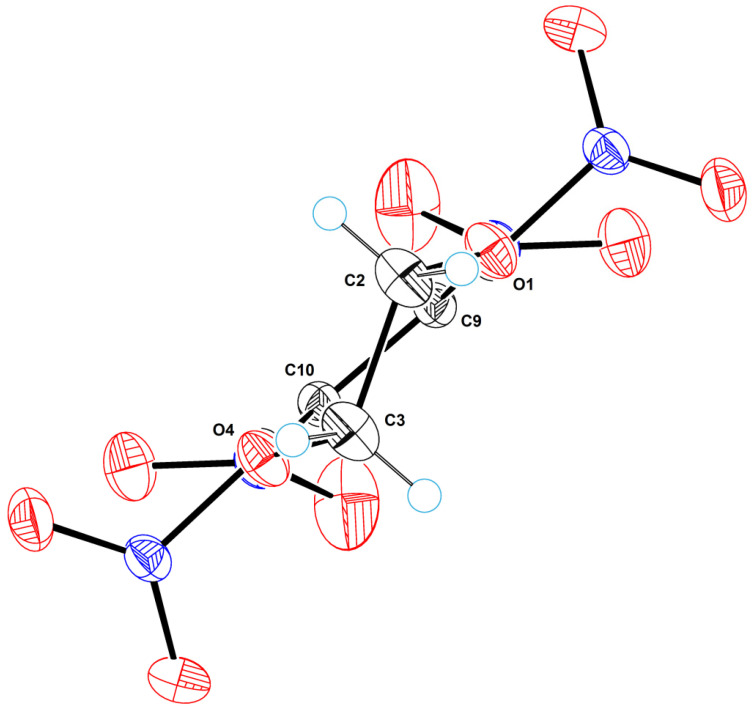
A projection of the molecular structure of TNBD along the twofold axis (carbon atoms are represented in black color, nitrogen—in blue and oxygen—in red).

In the crystal structure of TNBD, the molecules are in special positions; each molecule lies on the symmetry axis of order 2. Therefore, atoms O1 and O4, C2 and C3, C9 and C10, etc., are symmetrically equivalent. Figure 8 shows a projection of the molecule along the axis of symmetry. This figure clearly indicates that the dioxane ring in TNBD is characterized by a twist conformation. The exit of the C2 atom from the planar phenyl ring of the molecule is equal to 0.384(2) Å (correspondingly, the exit of the C3 atom is −0.384(2) Å). The dihedral angle between the phenyl ring of the molecule and the “triangle” O1–C2–C3 is 41.8(1)° (due to symmetry, the same angle is observed between the phenyl ring and the “triangle” C2–C3–C4). This nonplanar projection of the TNBD structure is shown in Figure 8.

**Figure 9:**
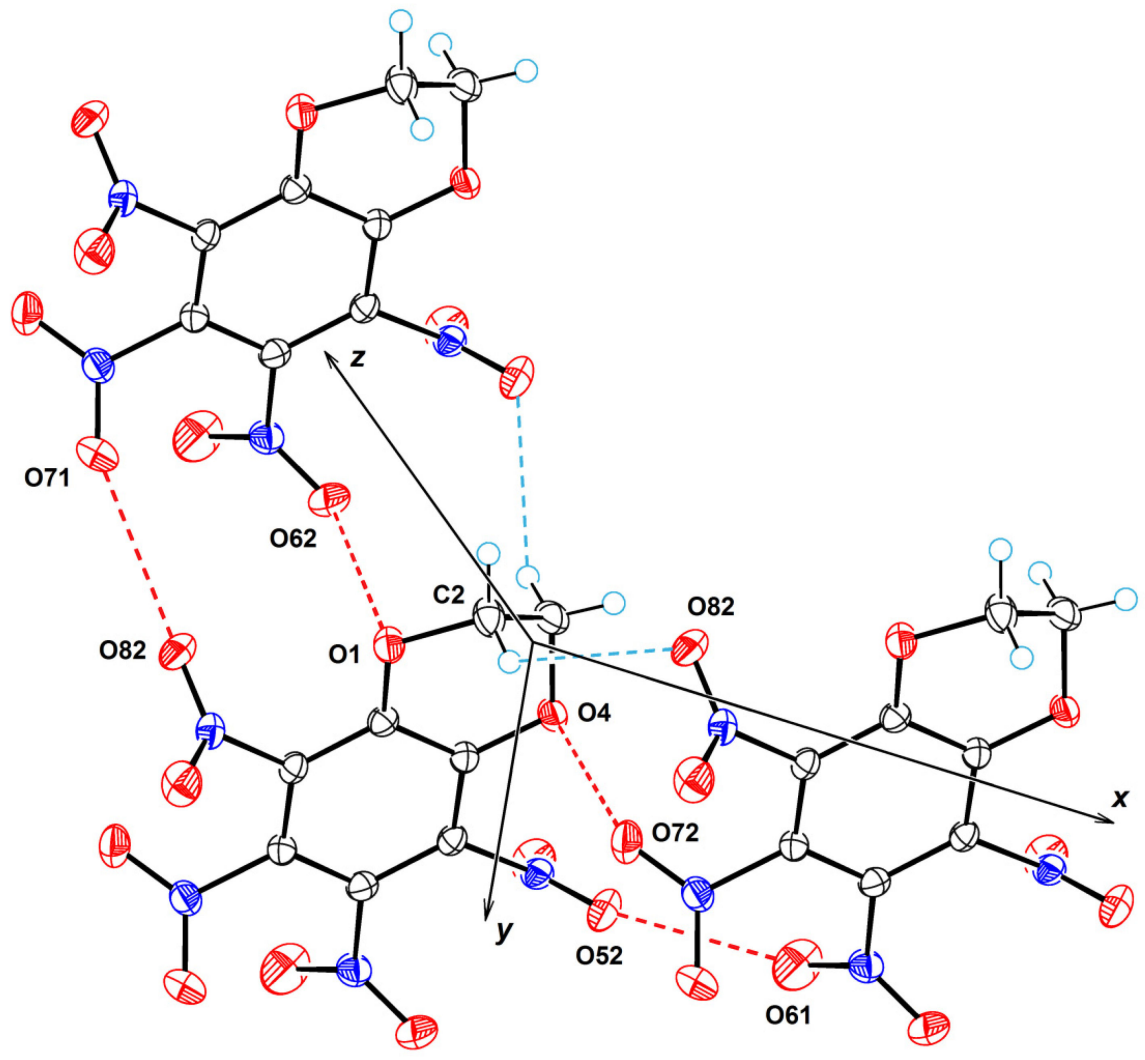
The formation of a molecular chain in the crystal structure of TNBD shows sigma-hole interactions and H-bonds (carbon atoms are represented in black color, nitrogen—in blue and oxygen—in red).

The packing of TNBD molecules in the crystal lattice is quite dense. The packing index, calculated using Kitaigorodsky’s approach [55,56], is equal to 0.745. The crystal structure is characterized by strong intermolecular sigma-hole interactions (see Figure 9). The distance between the O52 and O61 atoms of the neighboring molecules is 2.910(2) Å (the same distance occurs between the atoms O82 and O71 due to symmetry). Also noteworthy is the strong interaction between atoms O1 and O62 (as well as atoms O4 and O72), which is characterized by a distance of 2.910(2) Å. In addition, the carbon atom C2 (like C3) has increased electronegativity because it is bonded to oxygen. This promotes the formation of the intermolecular hydrogen bonds of CH···O type (see in Figure 9). The parameters of this H-bond are the following: C2···O82 = 3.321(3) Å, H2B···O82 = 2.75(2) Å and C2-H2B···O82 = 122(3)°; i.d., this bond is quite weak. Nevertheless, it participates in stabilizing the crystal structure. By means of these intermolecular interactions and hydrogen bonds, the molecular chains along the long parameter of the lattice are formed in the crystal structure. The consequence of these features of the crystal structure of TNBD is a significant packing index value and, in accordance with this, a quite high density of the substance.

A molecular chain formation in the crystal structure of TNBD demonstrating sigma-hole interactions and H-bonds is shown in Figure 9.

#### 2.1.6. Electrostatic Potential Map (MEP) for TNBD Molecule

**Figure 10:**
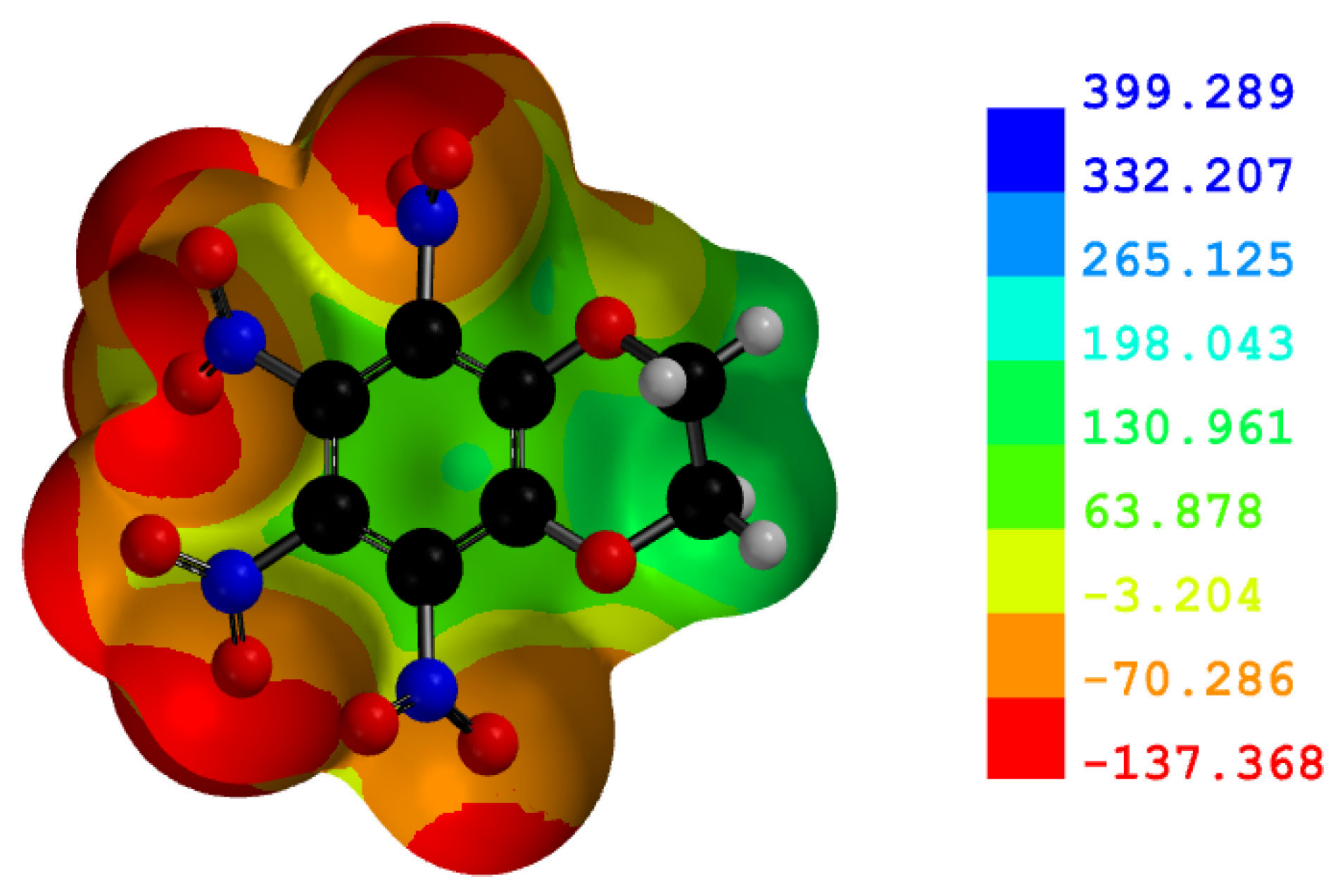
The surface of the electrostatic potential (MEP) of TNBD, calculated by the DFT-B3LYP/6-311G(d,p) method. The colors red and blue depict the compound’s negatively and positively charged regions (MEP isovalue = 0.002).

The initial refinement of the TNBD geometry was performed by the semi-empirical (PM6) method, and further optimization and calculation were performed by the DT-B3LYP functional method in conjunction with the 6–311 G(d,p) basis set. The compounds’ structure (shown in Figure 10) was globally optimized with symmetry restriction, and their local minima were characterized by the harmonic frequency analysis.

#### 2.1.7. Main Calculated Detonation Performance Parameters of TNBD in Comparison with Known Energetic Materials Possessing Close Structural Patterns

The main calculated detonation parameters of TNBD (at density 1.85 g/cm^3^) were compared to the known characteristics of the standard energetic compounds DNAN, TNAN, TNT and Tetryl as shown in Table 2.

The calculated enthalpy of the TNBD formation was estimated as −382 kJ/M (HF) and the detonation heat as −5112.9 kJ/M. The main parameters calculated for TNBD by EXPLO 5 [57,58,59] are represented below.

**Table 2:** The main calculated detonation parameters of TNBD (at density 1.85 g/cm^3^), compared to the known energetic materials.

Detonation Parameters	TNBD	DNAN, 2,4-dinitro- anisole [2]	TNAN, 2,4,6-Trinitro- anisole [2]	TNT [1]	Tetryl [1]
Oxygen balance, %	−40.48	−96.90	−62.50	−73.97	−47.40
Detonation temperature (K)	3874	2743	2366	3744	3370
Detonation pressure (kbar)	277.84	159	205	202	260
Detonation velocity (m/s)	7726.9 at d = 1.85 g/cm^3^	5706 at d = 1.341 g/cm^3^	6800 at d = 1.57 g/cm^3^	6950 at d = 1.64 g/cm^3^	7570 at d = 1.71 g/cm^3^
Volume of detonation products (L/kg)	555.8	626	760	730	800

These data demonstrate that the calculated detonation velocity (D) of TNBD is somewhat greater than that of the well-known energetic tetranitrocompound tetryl [1] (7727 vs. 7570 m/s) and similarly for the detonation pressure (P) (278 vs. 260 kbar (exp. [3]), whereas the energetic properties of TNBD in terms of the aforementioned parameters significantly outperformed DNAN, TNAN and TNT.

The primary advantages of TNBD over similar structural features (DNAN, TNAN and Tetryl) are its high density ρ (g/cm^3^) (TNBD > Tetryl > TNAN > DNAN), the best oxygen balance (OB_CO2_, %): (TNBD > Tetryl > TNAN > DNAN) and the detonation velocity (VOD, m/s) (TNBD > Tetryl > TNAN > DNAN). TNBD has only one major disadvantage: the high expense of preparation.

## 3. Materials and Methods

### 3.1. General Methods

All chemicals and solvents for the synthesis of the benzodioxane derivatives were from Sigma Aldrich (St. Louis, MO, USA). NMR spectra were recorded on Varian Unity Inova 300 spectrometer (Agilent Technologies, Santa Clara, CA, USA) (300 MHz) with tetramethylsilane (TMS) as internal standard. Chemical shifts were reported in ppm (δ). Infrared spectra recorded for KBr discs on Perkin-Elmer Spectrum GX FT-IR (Benelux Scientific BV, Leuven, Belgium). Raman spectra registered on FT-Raman spectrometer Horiba HR800 (HORIBA Europe GmbH, Darmstadt, Germany) for the 633 nm excited spectra. Melting points determined on capillary melting point apparatus Melt-Temp. Thermogravimetric analysis performed on LINSEIS STA PT 1600, Thermogravimetric Analyzer (Linseis Messgeraete GmbH, Selb, Germany). Quantum mechanical calculation was carried out using PC Spartan ‘10 (Wavefunction Inc., Tokyo, Japan, version 1.1.0). Single crystal X-ray diffraction evaluated using instrument “Bruker-Nonius KappaCCD”(Bruker Nonius, Delft & MacScience, Tokyo, Japan) (computing data collection “KappaCCD”) and computing data reduction “Denzo and Scalepak (Otwinowski & Minor, 1997)” were applied. All diagrams and calculations performed using maXus (Bruker Nonius, Delft & MacScience, Tokyo, Japan).

### 3.2. Optimized Synthesis of TNBD by Nitration of 6,7-dinitro-1,4-benzodioxane (6,7-dinitro-2,3-dihydro-1,4-benzodioxine)

**Scheme 1:**
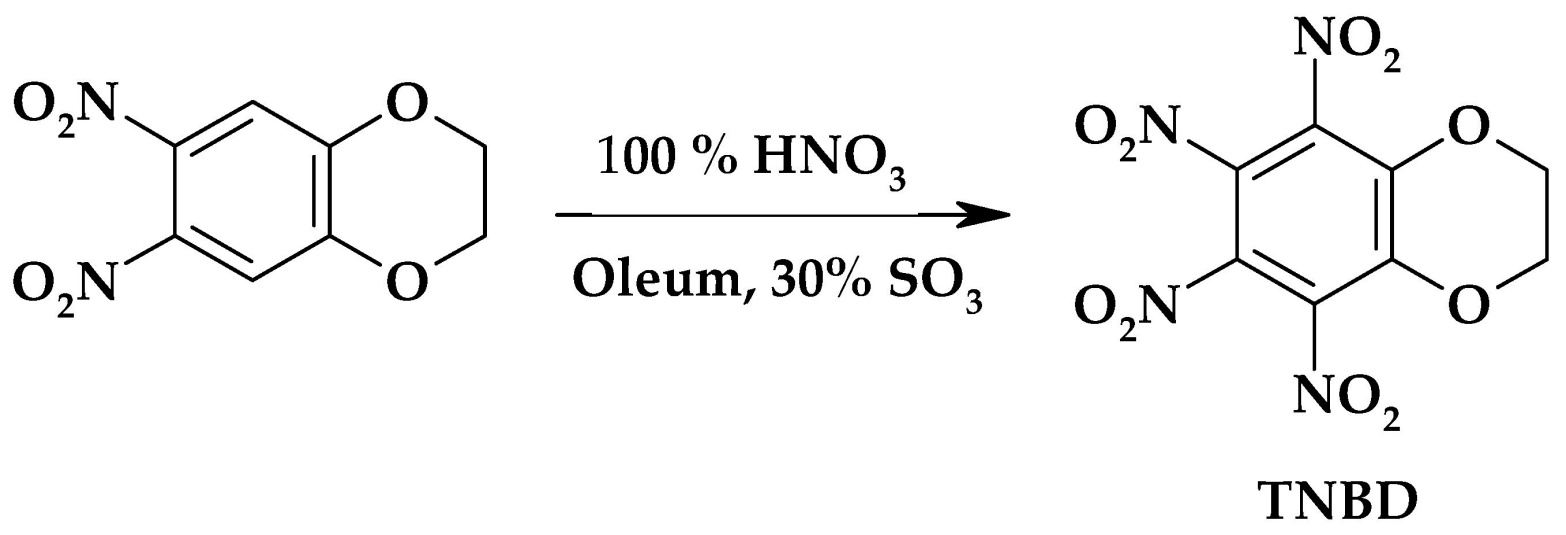
Synthesis scheme of TNBD.

6,7-Dinitro-2,3-dihydro-1,4-benzodioxine (prepared according to the procedure [60]) (25 g, 0.11 M) was added in small portions to the stirred 100% HNO_3_ (100 mL, 1.05 M) and cooled up to 10 °C. The reaction mixture was allowed to warm by gradually increasing the temperature from 10 to 70 °C through external heating in a water bath. After cooling to 40–50 °C, 50 mL of 98% H_2_SO_4_ was added by drops, and stirring was continued for 30 min. at 70 °C. The reaction was finalized by adding 60 mL of fuming sulfuric acid, containing 30% SO_3_, and stirring for 2 h at 80 °C (Scheme 1). After cooling to room temperature, the reaction mixture was poured into 0.5 kg of broken ice.

The supernatant was filtered, washed several times with cold distilled water and dried at room temperature. Yield: 28 g, 81%. The analytical sample of TNBD was obtained after two re-crystallizations with acetone. M. p. 286–288 °C (lit. m.p. 286 °C [61]). It must be noted that this optimized synthesis gave a better yield than our two previously tested variants: starting from 6-nitro-2,3-dihydro-1,4-benzodioxine (63%), or from 5,7-dinitro-2,3-dihydro-1,4-benzodioxine (71%), and using analogous reaction conditions.

NMR spectra: ^13^C spectrum (δ, [ppm, d_6_-DMSO, freq.–75 MHz): 143.91; 132.43; 129.02; 66.31; ^1^H spectrum (d_6_-DMSO, freq.–300 MHz): 4.73 (s, 4H).

FT-IR spectrum (cm^−1^): 2967, 2889, 1597, 1573, 1566, 1558, 1492, 1442, 1382, 1342,1335, 1309, 1249, 1230, 1078, 995, 951, 902, 860, 838, 797, 778, 726, 710, 654, 628, 438.

## 4. Conclusions

This study leads to the following conclusions:

TNBD (5,6,7,8-tetranitro-1,4-benzodioxane or 5,6,7,8-tetranitro-2,3-dihydro-1,4-benzodioxine, molecular formula—C_8_H_4_N_4_O_10_) has been synthesized in 81% yield by the nitration of 6,7-dinitro-1,4-benzodioxane.

Interestingly, TNBD has been found to have good chemical stability and can be stored under normal conditions for 20 years without displaying any signs of degradation (preliminary results according to the chromatographic (TLC) and spectral data), but a more detailed investigation on TNBD stability in the future is required. The hydrolytic stability was confirmed by an additional experiment when a sample of TNBD was stored under a water layer for 168 h at room temperature and any hydrolytic changes in the material were revealed (m.p., NMR sp.).

TNBD shares structural similarities with well-known energetic materials 2,4-dinitroanisole (DNAN) and 2,4,6-trinitroanisole (TNAN) and might be seen as their cyclic, more oxygen-rich and more powerful analogue. Furthermore, due to its higher density, it may be a better choice for high-density and high-energy materials (HDEMs).

The spectral characteristics, crystal structure and experimental density of TNBD (1.85 g/cm^3^) were investigated.

There are only a few hydrogen atoms in the structure of TNBD, but there are also significant sigma-hole interactions that contribute to its increased density. Another factor is the formation of intermolecular hydrogen bonds of the CH···O type.

All these factors are contributing to the increasing packing density of TNBD molecules.

The results of the thermal analysis (TGA) obtained from the twice re-crystallized material revealed the onset at 240 °C (partial sublimation started) and melting at 286 °C. The investigated material degraded completely at 290–329 °C, indicating that the thermostability of TNBD is very close to that of HNS (hexanitrostilbene), also known as a thermostable HEM [19].

The main detonation parameters were computed using M. Sućesca—EXPLO 5 program code.

The detonation velocity and detonation pressure of TNBD were found to be significantly higher than those of the standard aromatic energetic tetranitrocompound tetryl (*N*-methyl-*N*,2,4,6-tetranitroaniline), as well as those of other known energetic materials with similar structural patterns, such as TNT (2,4,6-trinitrotoluene), DNAN (2,4-dinitroanisole) and TNAN (2,4,6-trinitroanisole) [2].

To summarize all these data, TNBD appears to be a promising energetic compound that deserves further in-depth research in the future.

## Data Availability

Data are contained within the article and Supplementary Materials.

## Supplementary Materials

The following supporting information can be downloaded at: https://www.mdpi.com/article/10.3390/ijms25105099/s1.
