# Supplementary material for: Convenient Preparation, Thermal Properties and X-ray Structure Determination of 2,3-Dihydro-5,6,7,8-tetranitro-1,4-benzodioxine (TNBD): A Promising High-Energy-Density Material
Source: Int J Mol Sci. 2024 May 7;25(10):5099. doi: 10.3390/ijms25105099 (PMC11121466; doi:10.3390/ijms25105099)
Supplement: Supplementary file 1 [file ijms-25-05099-s001.zip › ijms-2962764-supplementary.pdf]

Supporting Information for

Communication

Convenient Preparation, Thermal Properties and X-ray Structure Determination of 2,3-Dihydro-5,6,7,8-tetranitro-1,4-benzodioxine (TNBD): A Promising High-Energy-Density Material

Jonas Šarlauskas

Department of Xenobiotics Biochemistry, Institute of Biochemistry of Vilnius University, Sauletekio 7, LT-10257 Vilnius, Lithuania; jonas.sarlauskas@bchi.vu.lt

Table S1: X-ray difraction analysis data. Geometry tables for the compound TNBD.

INTRAMOLECULAR BOND LENGTHS

Minimum bond length= 0.80Å : Maximum bond length= 1.60Å

O(1)	-	C(9)	1.338(2)	O(1)	-	C(2)	1.453(2)
O(82)	-	N(8)	1.212(2)	O(72)	-	N(7)	1.212(2)
O(81)	-	N(8)	1.225(2)	N(7)	-	O(71)	1.215(2)
N(7)	-	C(7)	1.473(2)	N(8)	-	C(8)	1.472(2)
C(7)	-	C(7)	1.383(3)	C(7)	-	C(8)	1.388(2)
C(9)	-	C(9)	1.405(3)	C(9)	-	C(8)	1.391(2)
C(2)	-	C(2)	1.503(3)	C(2)	-	H(2A)	0.92(3)
C(2)	-	H(2B)	0.91(2)				

INTRAMOLECULAR BOND ANGLES

Minimum bond length= 0.80Å : Maximum bond length= 1.60Å

C(9)	-	O(1)	-	C(2)	113.81(11)	O(72)	-	N(7)	-	O(71)	126.22(14)
O(72)	-	N(7)	-	C(7)	116.99(12)	O(71)	-	N(7)	-	C(7)	116.79(12)
O(82)	-	N(8)	-	O(81)	126.22(13)	O(82)	-	N(8)	-	C(8)	117.17(12)
O(81)	-	N(8)	-	C(8)	116.61(13)	N(7)	-	C(7)	-	C(7)	121.79(8)

N(7) - C(7) - C(8) 118.94(13) C(7) - C(7) - C(8) 119.18(8)

O(1) - C(9) - C(9) 122.69(7) O(1) - C(9) - C(8) 118.69(12)

C(9) - C(9) - C(8) 118.60(8) N(8) - C(8) - C(7) 120.19(12)

N(8) - C(8) - C(9) 117.51(12) C(7) - C(8) - C(9) 122.21(14)

O(1) - C(2) - C(2) 110.23(11) O(1) - C(2) - H(2A) 108.(2)

O(1) - C(2) - H(2B) 105.6(15) C(2) - C(2) - H(2A) 106.(2)

C(2) - C(2) - H(2B) 111.9(14) H(2A) - C(2) - H(2B) 115.(2)

INTRAMOLECULAR TORSION ANGLES (H omitted)

Minimum bond length= 0.80Å : Maximum bond length= 1.60Å

C(2) - O(1) - C(9) - C(9) 167.41(11) C(2) - O(1) - C(9) - C(8) 165.99(13)

C(9) - O(1) - C(2) - C(2) -136.50(10) O(72) - N(7) - C(7) - C(7) 43.76(9)

O(72) - N(7) - C(7) - C(8) 47.05(10) O(71) - N(7) - C(7) - C(7) -135.42(12)

O(71) - N(7) - C(7) - C(8) -132.12(14) O(82) - N(8) - C(8) - C(7) -113.15(13)

O(82) - N(8) - C(8) - C(9) 70.38(11) O(81) - N(8) - C(8) - C(7) 67.25(11)

O(81) - N(8) - C(8) - C(9) -109.22(12) C(7) - C(7) - N(7) - O(72) -136.24(14)

N(7) - C(7) - C(7) - N(7) 0.00(8) C(7) - C(7) - N(7) - O(71) 44.58(11)

N(7) - C(7) - C(7) - C(8) -176.70(13) C(8) - C(7) - N(7) - O(72) 47.05(10)

C(8) - C(7) - N(7) - O(71) -132.12(14) N(7) - C(7) - C(8) - N(8) 0.40(9)

N(7) - C(7) - C(8) - C(9) 176.7(2) C(8) - C(7) - C(7) - N(7) 176.70(12)

C(7) - C(7) - C(8) - N(8) -176.39(15) C(7) - C(7) - C(8) - C(9) -0.09(10)

C(8) - C(7) - C(7) - C(8) 0.00(8) O(1) - C(9) -
C(9) - O(1) 0.00(7)

O(1) - C(9) - C(9) - C(8) 178.59(13) C(9) - C(9) -
O(1) - C(2) -12.59(9)

O(1) - C(9) - C(8) - N(8) -1.20(8) O(1) - C(9) -
C(8) - C(7) -177.6(2)

C(8) - C(9) - O(1) - C(2) 165.99(13) C(8) - C(9) -
C(9) - O(1) -178.59(12)

C(9) - C(9) - C(8) - N(8) 177.44(14) C(9) - C(9) -
C(8) - C(7) 1.05(10)

C(8) - C(9) - C(9) - C(8) 0.00(8) C(7) - C(8) -
N(8) - O(82) -113.15(13)

C(7) - C(8) - N(8) - O(81) 67.25(11) N(8) - C(8) -
C(7) - N(7) 0.40(9)

N(8) - C(8) - C(7) - C(7) 3.61(8) N(8) - C(8) -
C(9) - O(1) -1.20(8)

C(9) - C(8) - N(8) - O(82) 70.38(11) C(9) - C(8) -
N(8) - O(81) -109.22(12)

N(8) - C(8) - C(9) - C(9) -2.56(7) C(7) - C(8) -
C(9) - O(1) -177.6(2)

C(9) - C(8) - C(7) - N(7) 176.7(2) C(9) - C(8) -
C(7) - C(7) 179.90(13)

C(7) - C(8) - C(9) - C(9) -178.95(13) O(1) - C(2) -
C(2) - O(1) -0.02(7)

C(2) - C(2) - O(1) - C(9) 43.50(10)

INTERMOLECULAR NON-BONDED DISTANCES

Minimum distance= 1.95Å : Maximum distance= 3.50Å

Atom(1)		Atom(2)	distance	ns	np	Ta	Tb	Tc	x(2)	y(2)	z(2)
O(1)	-	O(82)	3.0534(15)	3	1	0	0	1	0.19772	0.08779	0.59972
O(1)	-	O(71)	3.235(2)	2	1	0	1	1	-0.03531	0.22086	0.61436
O(1)	-	N(8)	3.448(2)	3	1	0	0	1	0.29370	0.04775	0.56425
O(82)	-	H(2A)	3.03(3)	3	1	0	0	1	0.42330	0.46537	0.67214
O(82)	-	H(2B)	3.25(2)	3	1	0	0	1	0.53069	0.38884	0.62140
O(72)	-	O(81)	3.388(2)	3	1	0	1	1	0.31223	1.00975	0.45335

O(72) - C(7)	3.457(2)	5	1	0	0	0	-0.05135	0.57427 0.20630
O(81) - C(7)	3.262(2)	2	1	0	1	1	-0.05135	0.42573 0.70630
O(81) - C(8)	3.220(2)	6	1	0	1	0	0.10141	0.54512 0.83681
O(81) - C(2)	3.461(2)	3	1	0	0	1	0.47263	0.39265 0.68007

----

ns is the symmetry operator number - (* denotes inversion indicator)

np is the lattice point number

DIHEDRAL ANGLES FORMED BY LSQ PLANES

PLANE - PLANE ANGLE E.S.D

PLANE 1 (C5 C6 C7 C8 C9 C10); PLANE 2 (O71 N7 O72); PLANE 3 (O81 N8 O82)

PLANE	PLANE	ANGLE	E.S.D
1	2	46.143	0.081
1	3	68.706	0.103
